# Wearable Sensors in Ambulatory Individuals With a Spinal Cord Injury: From Energy Expenditure Estimation to Activity Recommendations

**DOI:** 10.3389/fneur.2019.01092

**Published:** 2019-11-01

**Authors:** Werner L. Popp, Sophie Schneider, Jessica Bär, Philipp Bösch, Christina M. Spengler, Roger Gassert, Armin Curt

**Affiliations:** ^1^Spinal Cord Injury Center, Balgrist University Hospital, Zurich, Switzerland; ^2^Rehabilitation Engineering Laboratory, Department of Health Sciences and Technology, Eidgenössische Technische Hochschule (ETH) Zürich, Zurich, Switzerland; ^3^Exercise Physiology Lab, Department of Health Sciences and Technology, Eidgenössische Technische Hochschule (ETH) Zürich, Zurich, Switzerland

**Keywords:** energy expenditure, spinal cord injury, wearable sensor, estimation model, pathological gait, activity recommendation, digital biomarker

## Abstract

Inappropriate physical inactivity is a global health problem increasing the risk of cardiometabolic diseases. Wearable sensors show great potential to promote physical activity and thus a healthier lifestyle. While commercial activity trackers are available to estimate energy expenditure (EE) in non-disabled individuals, they are not designed for reliable assessments in individuals with an incomplete spinal cord injury (iSCI). Furthermore, activity recommendations for this population are currently rather vague and not tailored to their individual needs, and activity guidelines provided for the non-disabled population may not be easily translated for this population. However, especially in iSCI individuals with impaired abilities to stand and walk, the assessment of physical activities and appropriate recommendations for a healthy lifestyle are challenging. Therefore, the study aimed at developing an EE estimation model for iSCI individuals able to walk based on wearable sensor data. Additionally, the data collected within this study was used to translate common activity recommendations for the non-disabled population to easily understandable activity goals for ambulatory individuals with an iSCI. In total, 30 ambulatory individuals with an iSCI were equipped with wearable sensors while performing 12 different physical activities. EE was measured continuously and demographic and anthropometric variables, clinical assessment scores as well as wearable-sensor-derived features were used to develop different EE estimation models. The best EE estimation model comprised the estimation of resting EE using the updated Harris-Benedict equation, classifying activities using a k-nearest neighbor algorithm, and applying a multiple linear regression-based EE estimation model for each activity class. The mean absolute estimation error of this model was 15.2 ± 6.3% and the corresponding mean signed error was −3.4 ± 8.9%. Translating activity recommendations of global health institutions, we suggest a minimum of 2,000–3,000 steps per day for ambulatory individuals with an iSCI. If ambulatory individuals with an iSCI targeted the popular 10,000 steps a day recommendation for the non-disabled population, their equivalent would be around 8,000 steps a day. The combination of the presented dedicated EE estimation model for ambulatory individuals with an iSCI and the translated activity recommendations is an important step toward promoting an active lifestyle in this population.

## Introduction

“10,000 steps a day” is a popular activity recommendation which is understandable, easy to remember, and as a consequence easy to apply in daily life. Although this activity recommendation is not without controversy and other daily step goals have been proposed (1, 2), it is nonetheless a recommendation which is commonly used, also in consumer wearables, to promote a healthy lifestyle in the general population (3–5).

Large institutions such as the American College of Sports Medicine (ACSM), the U.S. Department of Health and Human Services (HHS), and the World Health Organization (WHO) present their activity guidelines as the duration per week that one should spend doing aerobic physical activity (PA) at a specific intensity, e.g., 150 min of moderate-intensity aerobic PA per week (6–9). While such activity guidelines are—from a scientific perspective—more elaborated than the 10,000 steps recommendation, they are more challenging to follow since the intensity, and duration of the activity need to be observed. While subjective intensity ratings, as proposed in the guidelines, or the average energy expended in a given activity, as presented by Ainsworth et al. (10, 11), can be used as estimates, it is easier and more precise to assess the intensity of a PA through measures or estimates of energy expenditure (EE). Commercially available (consumer- and research-grade) wearable activity trackers such as Fitbit One, Nike Fuelband, Jawbone UP, ActiGraph GT3X, or the Apple Watch series 3 (and higher), are used by non-disabled individuals to estimate EE in daily life. However, depending on the device, the estimation accuracy can range from poor to good (12–14). Thus, accurate wearable activity trackers have been shown to promote PA and an active lifestyle (15–17).

Only 13–16% of the population with a spinal cord injury (SCI) reports being physically active (18), while increased odds of heart disease have been reported for this population (19). Furthermore, greater levels of PA were associated with lower levels of cardiovascular diseases and type 2 diabetes risk factors (20). Therefore, wearable activity trackers could be of great value to increase the amount of PA and therefore reduce the risk of cardiometabolic diseases in this population. There exist different EE estimation models based on wearable sensor data for individuals with an SCI using a wheelchair, which can partly be applied in real-world applications (21–30). However, to the best of our knowledge, no such EE estimation model exists for ambulatory individuals with an incomplete SCI (iSCI) where the suitability of models developed for ambulatory individuals needs to be questioned (31). General models for ambulatory individuals usually estimate EE using step counts and/or activity counts (AC), which is an approximate for the amount of limb movements. Four major problems exist in this context: First, the magnitude of AC largely depends on the device and is therefore not a robust metric. Second, step detection of commercial sensors used for persons with pathological gait can partly be poor, especially for persons with a low walking speed as shown for iSCI (31) and other neurological conditions (32, 33). Third, the EE during pathological gait increases with more severe gait disability (34–36), which is not taken into account by commercial sensors. Fourth, the use of upper extremity assistive devices, e.g., crutches and canes, increases the EE (34), which is not necessarily captured when estimating EE through step counts or AC. Nonetheless, it has been shown in other populations with altered or pathological gait (e.g., elderly, multiple sclerosis, and stroke) that EE can be estimated using wearable sensors (37–40). Furthermore, multi-sensor setups might provide a superior EE estimation accuracy compared to single-sensor setups as shown in neurological patients (41), since impairments manifesting at different body locations can be taken into account. Therefore, it can be assumed, that an accurate EE estimation model based on wearable sensor data can be developed for ambulatory individuals with an iSCI.

In addition to accurate activity tracking with EE estimation, this population also needs comprehensive activity recommendations adapted to the special needs. Although, general activity guidelines for this population exist (8, 9, 42, 43), there are no simple and comprehensive activity recommendations, which are applicable for iSCI and during acute rehabilitation (44) as for example known for the non-disabled population, e.g., 10,000 steps per day.

For that reason, we have conducted a study aiming at developing and validating an EE estimation model based on data from multiple wearable sensors for ambulatory individuals with an iSCI. Furthermore, we aimed at translating activity recommendation for the non-disabled population to comprehensive recommendations for ambulatory individuals with an iSCI. The study design, the data analysis, and the translation of activity recommendations were inspired by a previous study from our group (30), which focused on wheelchair-dependent SCI individuals with the same aims as this study.

## Methods

As the present study is closely related to a study we have conducted with SCI wheelchair users (30), some parts of the methods section are almost identical to the previous study. To facilitate comprehension, we nevertheless provide detailed methodological information here.

### Participants

Thirty participants with iSCI (age 54.1 ± 11.9 years, 10 tetraparetic, 20 men) and some ability to stand and walk were recruited for this study. Participants had to be over 18 years old and the SCI had to be in the chronic stage (>1 year post-injury). Furthermore, participants with all neurological levels of injury (NLI) as well as levels of impairment according to the ASIA Impairment Scale (AIS) from B to D were admitted to the study. For inclusion, participants had to be able to walk at least 100 m without supervision. Any neurological disease other than SCI affecting upper or lower limb function and any orthopedic, rheumatologic, or metabolic disorders were considered an exclusion criterion. The local ethics committee of the canton of Zurich (KEK-ZH Nr. 2013-0202) approved the study and participants provided written informed consent in accordance with the declaration of Helsinki before participating. Demographic information about the participants can be found in Table 1. Five participants depended on walking aids in order to complete our study. Two participants used forearm crutches, one used walking poles, one used a rollator, and one had two lower-limb orthoses and used forearm crutches.

**Table 1:** Demographics and assessment scores (SCIM III sub-section mobility and 6MWT) of all participants.

**Variables**	**Values**
Participants	30
Sex	
Male	20
Female	10
Age (years)	54.1 ± 11.9 (27–72)
Weight (kg)	75.5 ± 16.2 (44–106)
Height (m)	1.71 ± 0.09 (1.48–1.91)
Injury level	
C2-T1	10
Th2-L4	20
AIS score	
B	1
C	1
D	28
SCIM III mobility	28.1 ± 3.9 (15–30)
6MWT (m)	486 ± 158 (137–744)
Reported hours of	3.9 ± 3.6 (0–14)
Sport/week	

*Note that age, weight, height, SCIM III mobility score, 6MWT distance, and reported hours of sports per week are presented as mean ± standard deviation (minimum—maximum)*.

### Measurement Device

#### Activity Monitor

**Figure 1:**
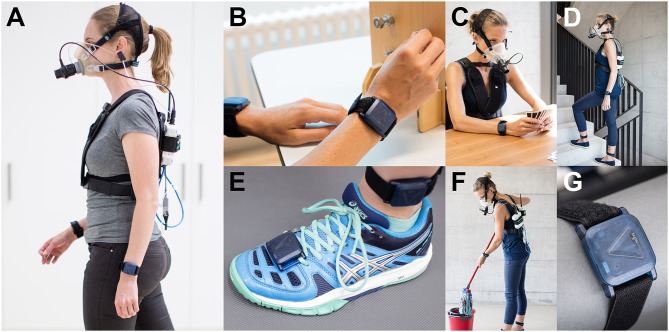
Examiners equipped with the full measurement setup, i.e., eight JUMP modules and the indirect calorimeter. **(A)** Side view of an examiner during the activity walking with two JUMP modules attached at each wrist and the indirect calorimeter with the facemask (with a turbine), data exchange unit, and the sensor box. Note, that the JUMP modules placed at the chest and the hip are not visible (usually hidden under the shirt). **(B)** Extract from the GRASSP assessment during the prehension part. **(C)** Examiner wearing the experimental setup during the activity playing cards and **(D)** climbing stairs. **(E)** Close view of the JUMP modules fixed at the foot and at the ankle. **(F)** Examiner during the activity sweeping a mop. **(G)** Close view of the JUMP module recently developed by the ZurichMOVE consortium. Written and informed consent was obtained for the publication of these images.

A novel 10-degrees-of-freedom inertial measurement unit (IMU), which was developed by the ZurichMOVE consortium (www.zurichmove.ch), was used for this study. The so-called JUMP (Joint University Motion Platform) module (Figure 1) comprises a motion processing unit (MPU-9250, InvenSense Inc., San Jose, CA, USA) including a 3-axis accelerometer, a 3-axis gyroscope, and a 3-axis digital compass. Additionally, it includes a separate 3-axis high-g accelerometer (ADXL375, Analog Devices Inc., Norwood, MA, USA), an altimeter (MS5611-01BA01, TE Connectivity Ltd., Schaffhausen, Switzerland), a microcontroller (STM32L476RG, STMicroelectronics, Geneva, Switzerland), a custom made PCB, a 4 GB microSD card for data storage, and a rechargeable lithium ion polymer battery. For wireless sensor synchronization, the JUMP module further comprises a Bluetooth® low energy system-on-chip (nRF51822, Nordic Semiconductor, Trondheim, Norway). A robust and biocompatible 35 × 35 × 12 mm housing encloses the electronics. The JUMP module can continuously record data for 65 h at a sampling rate of 200 Hz (>72 h at 50 Hz) while synchronizing the modules in real time. Data transfer to the PC, battery charging, as well as sensor configuration are done through the corresponding docking station. Data in this study were collected with a sampling frequency of 200 Hz.

#### Metabolic Assessments

For the measurement of EE, a portable metabolic cart (Oxycon Mobile, Carefusion, Hoechberg, Germany) was used (Figures 1A,C,D,F). The system was calibrated 30 min prior to an experimental session and after each experimental session. For the extraction of EE, the proprietary software JLAB (Carefusion, Hoechberg, Germany) was used. For more information about the Oxycon Mobile, the reader is referred to our previous work (30).

### Clinical Assessments

Six clinical assessments were performed prior to, during, or after the experiment. The International Standard for Neurological Classification of Spinal Cord Injury (ISNCSCI) protocol was used to determine the NLI as well as the level of impairment, i.e., AIS grade (45). In addition, the Spinal Cord Independence Measure III (SCIM III) was used to assess the level of independence (46) and the Graded and Redefined Assessment of Strength, Sensibility and Prehension (GRASSP) was used to assess the sensorimotor hand function (47, 48) (Figure 1B). In order to assess locomotor function and gait, the 10-m Walk Test (10MWT) and the 6-Min Walk Test (6MWT) were used (49). The Timed Up and Go Test (TUG) was used to additionally assess balance and fall risk (49).

### Activities/Tasks

Participants were asked to conduct 12 different physical activities during this experiment. These activities were pseudo-randomly selected from a comprehensive pool of 34 activities of daily living. The pool covered activities related to rest, leisure time (e.g., watching TV and playing cards, Figure 1C), housework (e.g., washing dishes and sweeping with a mop, Figure 1F), office work (e.g., writing and computer work), sport and fitness (e.g., playing table tennis and doing squats), as well as locomotion (e.g., walking at given speed and climbing stairs, Figure 1D). These activities were further separated into four activity classes, namely “sedentary,” “low-intensity,” “high-intensity,” and “walking.” All activity classes with the associated physical activities are presented in the results section (**Figure 5**). Note, that the activity “weight lifting” was performed on a dip machine with 5 kg weights. During the activities including the bicycle ergometer, the resistance was set to 1, 1.25, and 1.5 W per kg of body weight. All “walking” and “running” activities with a given speed were performed on a straight 65 m track (indoor), where the speed was imposed through acoustic cues, i.e., participants had to reach a mark (5 m interval) always at the beep. The obstacle parcour consisted of four elements, two of them, namely the ramp and the stairs, were obstacles from the Cybathlon 2016 (50), one element was a slalom with six pylons, and the last element was a 360° rotation around a pylon. One lap was ~20 m long.

### Protocol

The protocol is almost identical to the one we used in the study with wheelchair users (30). After an overnight fast, participants came to the Balgrist University Hospital for one single session lasting around 5 h. Participants were not allowed to perform vigorous exercise and consume alcohol 24 h prior to the measurement. In the first step, the experimental procedures, as well as the 12 pseudo-randomly selected tasks, were explained in detail to the participants. Note that activities, which could not be performed by the subject, were replaced by an activity of the same activity class. Following the explanations, participants were equipped with eight sensors (Figure 1). Two sensors were attached to each wrist, similar to a watch, two sensors were attached ~5–10 cm above each ankle on the lateral side, two sensors were attached to the top of each foot (location: above the 3rd and 4th metatarsal bones), one sensor was attached to the chest above the sternum, and one sensor was attached above the right anterior superior iliac spine, similar to a pedometer attached to the belt. Furthermore, participants were equipped with an indirect calorimeter, i.e., Oxycon mobile.

The actual experimental session started with a measurement of resting EE (REE) lasting 20 min where participants had to lie on a bed without moving. Thereafter, participants received a standardized breakfast, which corresponded to ~450 kcal. During the following ≥90-min resting period (to ensure that REE had returned to baseline prior to further testing), clinical assessments were performed and the demographic, anthropometric, and behavioral data (age, height, weight, gender, handedness, and sportive activities per week) were collected. After the resting period, the experimental session continued with 12 different activities. The first activity was always lying on the bed for 20 min. This was considered as a second REE measurement under non-fasting condition to check whether the REE returned to the baseline. An additional break was added in case the REE did not return to the baseline. Thereupon, participants performed the pseudo-randomly selected, remaining 11 activities for 8 min each with a 5-min break following each of the activities. Activities were executed according to their expected intensity, starting with the least intense activity. Perceived exertion of each activity was rated by the participant on an 11-point numeric rating scale (0 = “no exertion,” 10 = “maximum exertion”) immediately after finishing the activity. Additionally, video recordings (GoPro Hero3, Go Pro Inc., San Mateo, CA, USA) were taken for the entire experimental session, including the clinical assessments.

A shortened protocol was performed by 13 participants. This required participants to come to the laboratory at least 2 h after the last food intake. The experimental session started with the clinical, demographic, anthropometric, and behavioral assessments followed by a single 20 min REE measurement. The following activity protocol was identical to the one described above.

### Data Analysis

**Figure 2:**
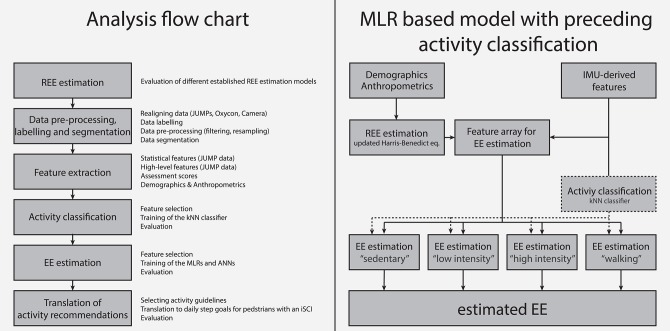
**(Left)** Analysis flow chart used in this study. **(Right)** Overview of the different analysis steps for the MLR based model with preceding activity classification.

The entire data handling was performed using MATLAB 2017b (The MathWorks, Natick, MA, USA). This included the merging of IMU data, Oxycon data, demographic, anthropometric and behavioral information, and assessment scores, the data preprocessing, the data labeling, and segmentation, the extraction of features, the training and evaluation of the different evaluation models, i.e., multi-linear regression model (MLR), k-nearest neighbor (kNN) classifier, artificial neural networks (ANN), as well as the statistical analysis. Furthermore, the calculations for translating activity recommendations were also performed using MATLAB 2017b. A brief overview of the different steps of processing and analysis can be found in Figure 2 (left).

In this study, we developed four different EE estimation models. Two models, namely an MLR-based and an ANN-based model, considered features derived from the IMU data, anthropometric data, clinical assessment data, as well as the estimated REE as direct input. The other two EE estimation models were motivated by the fact that it has been shown that preceding activity classification can significantly increase the EE estimation accuracy (30, 51, 52). Thereby, a PA is first classified into one of the four aforementioned activity classes, and afterwards a dedicated algorithm for each class is used to estimate the EE. This means that each activity class has its own EE estimation model (again MLR or ANN based). For illustration, the flow chart of the MLR based EE estimation model with a preceding activity classification is presented in Figure 2 (right). For each model, all features described above were considered as model inputs. However, in the end, the selected features differed between models (Table 2).

**Table 2:** Overview of the features selected for the different estimation models.

**Model description**			**Demographics**	**Statistical features**						**High-level features**	**Number of neurons**
MLR direct		REE^*^	-	MEAN(a_M_DW_)	PERC_1(a_M_Ch_)	MEAN(ω_M_DW_)	VAR(H_Ch_)	PERC_25(a_M_DA_)	VAR(a_M_NDF_)		n.A.
ANN direct		REE^*^	-	MEAN(a_M_Ch_)	MEDIAN(ω_Z_DA_)	PERC_25(a_M_DW_)	VAR(ω_M_DF_)				6
**Class Dependent Models**											
MLR	*Sedentary*	REE^*^	-	PERC_25(ω_M_Ch_)	AC(a_M_NDF_)					LAT (feet)	n.A.
	*Low-intensity*	REE^*^	-	SD(H_NDW_)	PERC_75(a_M_Hi_)	VAR(H_DF_)				LAT (feet)	n.A.
	*High-intensity*	REE^*^	-	PERC_3(ω_M_NDW_)	SD(H_NDW_)	PERC_5(a_M_Ch_)	RMS(a_M_DA_)	PERC_1(a_M_DF_)			n.A.
	*Walking*	REE^*^	weight	MEDIAN(a_M_Ch_)	PERC_1(ω_M_Hi_)	IQR(a_M_NDF_)	MEDIAN(ω_M_NDF_)				n.A.
ANN	*Sedentary*	REE^*^	-	PERC_25(ω_M_Ch_)	SD(H_Ch_)	PERC_3(a_M_DF_)					3
	*Low-intensity*	REE^*^	-	VAR(H_Ch_)	AC(a_M_Hi_)						3
	*High-intensity*	REE^*^	-	PERC_1(a_M_Ch_)	VAR(H_Ch_)	RMS(a_M_DA_)				CORR(feet)	3
	*Walking*	REE^*^	weight	PERC_25(a_M_Ch_)	VAR(ω_M_Hi_)	IQR(ω_M_Hi_)					3

Features were considered from the dominant (DW) and non-dominant (NDW) wrists, from the chest (Ch), from the hip (Hi), from the dominant (DA), and non-dominant (NDA) ankles, and from the dominant (DF) and non-dominant feet (NDF). From the statistical features, the mean (MEAN), median (MEDIAN), variance (VAR), standard deviation (SD), different percentiles (PERC_{1, 3, 25, 75}), root mean square (RMS), interquartile range (IQR), and activity counts (AC) were included. From the high-level features, the laterality (LAT) and the correlation between sensors (CORR) were included and in general, data was computed from the acceleration magnitude (aM), gyroscope magnitude (ωM), gyroscope Z-axis (ωZ), and from the altitude data (H).

* *Note that REE was included as a feature in all models*.

#### Estimation of REE

Different well-established and validated REE prediction equations for the non-disabled population were considered for the analysis. The Harris-Benedict equation (53), the updated Harris-Benedict equation (54), and the Mifflin-St Jeor equation (55) showed good results in a study with wheelchair users (30) and were therefore evaluated in this study. We hypothesized that these equations should show better estimates in iSCI able to stand and walk than in wheelchair users with SCI because muscle mass and body composition of participants with an iSCI are more similar to the one of the non-disabled population. We further included two additional estimation equations, namely the Müller equation and the body mass index (BMI)-dependent Müller equation (56). The criterion for selecting one of the equations for our REE estimation was the mean absolute error (MAE) in percent.

#### Data Pre-processing

First, the data were temporally realigned using a linear interpolation function if necessary. Afterwards, IMU data, Oxycon data, and video recordings were temporally aligned using dedicated markers, which were present in all data sets. Identical to the previous study with wheelchair users, we used a 2nd order Butterworth high-pass filter with a cutoff frequency of 0.25 Hz to filter the accelerometer data and a 2nd order Butterworth low-pass filter with a cutoff frequency of 0.2 Hz to filter the altitude data (30).

#### Labeling and Segmentation

Data was labeled using dedicated markers, which were available in both, IMU and Oxycon data. For REE, the average of EE between minute 14 and 18 of the first REE measurement was calculated. For the EE estimation and the activity classification, data was segmented in windows of 1 min. Only data from minute 4–8 of each activity were included in the analysis to ensure that EE reached a steady state. After the segmentation, data was checked visually for completeness. Segments with indirect calorimeter data and/or missing sensor data, segments in which EE had not reached a steady state, or in which participants did not follow the protocol (e.g., movements not related to the activity), were removed from the analysis. As a consequence, exactly 1,300 segments remained for the analysis. This corresponds to 90.3% of the entire data set.

For the translation of activity guidelines, the exact number of steps was required. Therefore, an investigator labeled all steps manually for all activities, which were included in this part of the analysis, i.e., walking at 1–6 km/h and walking at a self-chosen pace. Identification of individual steps was performed by labeling local peaks in the gyroscope signal and by checking the video recordings where applicable.

#### Feature Extraction

From the sensor data, we extracted two classes of features. First, we extracted statistical features, which were derived from one single sensor. Second, we extracted high-level features, which were either derived from multiple sensors or required multiple calculation steps. In total, 36 purely statistical features were derived for both wrist and feet sensors and for the hip and chest sensor. For both ankle sensors, 52 features were derived. These features were derived from the acceleration magnitude, from the gyroscope magnitude, from the gyroscope z-axis data corresponding to a rotation in the sagittal plane (only ankle sensors), and from the altitude data. The extracted features have already been used in other studies using machine learning for activity classification (23, 30, 57–64). Additionally, we included 12 high-level features. The number of steps was derived using an unpublished algorithm from our group, which is based on a peak detection algorithm applied to the gyroscope z-axis data of the ankle sensors. The distance walked was calculated by multiplying the number of steps by the step length, whereby the step length was estimated using the static method presented by Pratama and Hidayat (65). Based on the step detection, we also included the variance in stride time as a feature. Furthermore, limb-use laterality (66–68) for the upper limb (left vs. right wrist), lower limb (left vs. right ankle and left vs. right foot), as well as for upper limb (both wrists) vs. lower limb (both ankles) were included as additional features. The cross-correlation was computed for the accelerometer magnitudes of wrists (left vs. right), ankles (left vs. right), and feet (left vs. right). Finally, we included altitude change, activity counts (AC), all demographic and anthropometric variables, and all assessment scores as features (30, 69). The REE was also included as a feature but only for the EE estimation and not for the activity classification. Altogether, 351 features were available for the activity classification and the EE estimation. A list of all extracted features can be found in Table S1.

#### Activity Classification

The goal of the activity classification was to assign the different activities to one of the four activity classes, i.e., “sedentary,” “low-intensity,” “high-intensity,” and “walking.” The kNN classifier was used for the activity classification because it showed good results in previous studies from our group (30, 62, 63). For this classifier, we set the number of neighbors to be k = 10, and the squared inverse distance weight was used as the distance weighting function method. To evaluate the classification performance, we used the leave-one-subject-out cross-validation method, resulting in a total of 30 iterations. The percentage of correctly classified segments was used as a criterion for tuning and evaluating the classifier. Finally, five features were selected for the classifier, i.e., the 95th percentile (dominant ankle) and the interquartile range (IQR, dominant ankle) of the z-axis angular velocity signal, the 99th percentile (chest) and the 25th percentile (non-dominant wrist) of the angular velocity magnitude, and the standard deviation (SD) of the altitude signal (non-dominant wrist).

#### Estimation of EE

The four different EE estimation models presented in this study were based either on MLRs or on ANNs. The MLR models have all the basic form:

$$\begin{array}{l} EE=\;\beta_{0}+\overset{n}{\underset{i=1}{\sum }}\beta_{i}\cdot F_{i} \end{array}$$

with β_0_ representing the intercept and β_i_ representing the regression coefficient for the feature F_i_. The MLRs were computed by minimizing the sum of squared relative errors (70). This approach has been chosen because in this study, we used the MAE in percent as a criterion. Similar to the kNN classification, the performance was analyzed using leave-one-subject-out cross-validation, resulting in 30 iterations of training and testing. For the ANN-based approach, ANNs with one hidden layer with sigmoid neurons were used, whereby the number of neurons varied between three and six for the different models. The Levenberg-Marquardt backpropagation algorithm was used to train the algorithm, and the initial weights were chosen by the Nguyen-Widrow layer initialization function (71, 72). Again, the performance was evaluated using the leave-one-subject-out cross-validation and the MAE in percent. For training of the different ANNs, the data in each cross-validation iteration was further randomly divided into 70% training data and 30% validation data. The training process ended when 500 training epochs were reached, when the error gradient on the training set dropped below 1e-6, or when the error in the validation data set increased six times in a row. As the ANN output depends on the initial weights and the data division, 100 ANNs were trained per cross-validation iteration and the mean outcome was used for further analysis. Note that, for the two models (MLR and ANN) with preceding activity classification, EE estimation models had to be trained for each class separately. The two estimation models with preceding activity classification were evaluated in two ways. First, we evaluated the model assuming 100% correct activity classification and second, we used the output of the previously trained kNN classifier. An overview of the selected features of each EE estimation model can be found in Table 2.

#### Translation of Activity Recommendation

Different PA recommendations from global institutions were translated into daily step goals. The WHO and the U.S. Department of Health and Human Services (HHS) suggest 150 min of moderate-intensity aerobic PA per week or as an alternative 75 min of vigorous-intensity aerobic PA per week (7–9). As level walking is considered to be of low to moderate intensity, we only evaluated the moderate-intensity WHO recommendation. Similarly to the WHO and HHS recommendation, the ACSM and the American Heart Association (AHA) recommend a minimum of 30 min of moderate-intensity aerobic PA on 5 days per week (6). Other recommendations go one step further and include 30 min of moderate PA on every weekday (73, 74). For additional health benefits, most of the activity guidelines mentioned above recommend twice the amount of moderate PA per day (e.g., 60 min/day or 300 min/week) for additional health benefits, e.g., prevention of weight gain (74). Therefore, we translated the 30 and 60 min of moderate-intensity PA recommendation in a daily step goal for ambulatory individual with an iSCI. The basis for this translation was that a moderate-intensity PA of 60 min corresponds to an additional EE of 150–200 kcal (74). Furthermore, we also translated exercise guidelines for adults with an SCI. These guidelines suggest 20 min of moderate to vigorous-intensity aerobic exercise two times per week for cardiorespiratory fitness and 30 min of moderate to vigorous-intensity aerobic exercise three times per week for cardio-metabolic health benefits (42, 43). Thompson et al. (75) proposed that a PA of 150 min in moderate-to-vigorous intensity corresponds to ~1,000 min of total PA in moderate-to-vigorous intensity, which is easier to capture by wearable sensors. Therefore, we also translated this recommendation into a daily step goal.

Lastly, the activity goal of 10,000 steps a day is a common and easily understandable activity goal for the able-bodied population. Therefore, the daily goal of 10,000 steps was considered as a mutual benchmark for this analysis. For the translation of this activity goal, a value of 300 kcal [lower end of the suggested 300–400 kcal value by Choi et al. (76)] was used.

### Performance Analysis and Statistics

The MAE was used as a criterion for the EE estimation models. The REE and EE models were further analyzed using the MAE in kcal, the mean signed error (MSE) in percent and in kcal/day, and the maximum error in percent and kcal/day. In order to compare the result of the best model to results of the literature, we further computed Pearson correlation coefficient between estimated and measured EE. The performance of the kNN classifier was evaluated with the overall classification accuracy, which was used at the same time as a criterion. We used for the comparison of different activities the metabolic equivalent of task (MET) formula for the able-bodied population, where 1 MET represents 3.5 mL O_2_·kg^−1^·min^−1^ (10) and not the adapted formula for SCI. The correlation between perceived exertion and MET was computed by using a Spearman rank correlation.

A Bland-Altman analysis of agreement was performed between the measured EE and predicted EE using 95% limits of agreement. Additionally, a paired equivalence test was performed between the measured EE and the predicted EE with a chosen equivalence zone of ± 10% of the mean of the measured EE.

## Results

### REE Estimation

The evaluation of the five included REE estimation models can be found in Table 3. The different models were evaluated in terms of MAE, MSE, and maximum error and the criterion for the selection of one of the estimation models for the subsequent analysis was the MAE. Based on these results, the updated Harris-Benedict equation was selected for the further analysis, showing an MAE of 10.5 ± 9.4%. Note that in general, all models lay within 2.5% of MAE deviation. In all estimation models, the REE estimation was better for paraparetic participants compared to tetraparetic, and for women compared to men. Furthermore, all models tended to underestimate the REE. A plot showing the correlation between estimated REE using the updated Harris-Benedict equation and the measured REE can be found in Figure S1.

**Table 3:** Results of the resting energy expenditure (REE) estimation based on existing models from the literature.

**Model description**	**Mean absolute error (MAE)**						**Mean signed error (MSE)**		**Maximum error**	
	**Overall**		**Tetraparetic**	**Paraparetic**	**Men**	**Women**	**Overall**		**Overall**	
	**[%]**	**[kcal/day]**	**[%]**	**[%]**	**[%]**	**[%]**	**[%]**	**[kcal/day]**	**[%]**	**[kcal/day]**
Harris-Benedict^*^	10.8 ± 8.8	174 ± 131	14.0 ± 11.2	9.0 ± 6.7	11.5 ± 9.4	9.3 ± 7.6	−3.8 ± 13.5	−94 ± 199	42.9	528
updated Harris-Benedict^*^	10.5 ± 9.4	168 ± 132	13.9 ± 12.6	8.6 ± 6.7	11.2 ± 10.3	9.3 ± 7.7	−3.3 ± 13.9	−86 ± 198	47.9	534
Mifflin-St Jeor^*^	13.0 ± 9.8	216 ± 145	17.1 ± 12.7	10.7 ± 7.0	13.8 ± 10.2	11.5 ± 9.2	−7.9 ± 14.4	−162 ± 206	50.0	616
Müller^*^	11.1 ± 11.8	173 ± 140	14.4 ± 17.4	9.2 ± 6.8	11.5 ± 13.6	10.4 ± 7.5	−2.3 ± 16.2	−74 ± 212	64.3	550
Müller, BMI dependent^*^	11.0 ± 13.1	171 ± 151	14.9 ± 19.5	8.8 ± 7.1	12.0 ± 15.1	8.9 ± 8.2	−1.8 ± 17.1	−71 ± 219	71.5	612

As a criterion for choosing the best REE estimation model, the mean absolute error (MAE) in percent was used. Therefore, the updated Harris-Benedict equation was used for the subsequent analysis.

* *Equations known from the literature*.

### Activity Classification

**Figure 3:**
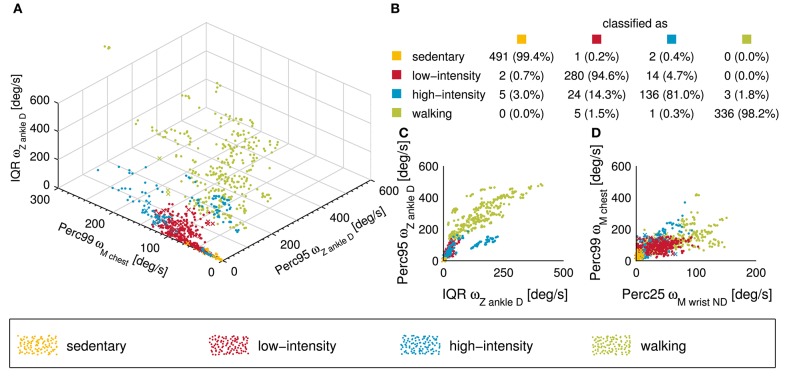
Visual presentation of the classification performance of the k-nearest neighbor (kNN) classifier used in this study. **(A)** 3D scatter plot showing the different activity classes for the three features explaining most of the variance. Note that a point corresponds to a correctly classified activity while a cross represents an incorrectly classified activity. **(B)** Confusion matrix of the entire data set (*n* = 1,300) is presented in this subplot. The overall classification accuracy of the kNN classifier was 95.6%. **(C,D)** Two 2D scatter plot showing the different activity classes from a different point of view.

The kNN classifier with the five previously mentioned inputs showed an overall classification accuracy of 95.6%. The sensitivity for the sedentary (99.4%) and walking class (98.2%) was excellent, for the low-intensity class (94.6%) very good, and for the high-intensity class (81.0%) good. The decreased classification accuracy of the high-intensity class results mainly from a misclassification into the low-intensity class (14.3%). The complete overview of the classification accuracies of each class and selected visualizations of the results can be found in Figure 3.

### EE Estimation

The results of the different EE estimation models can be found in Table 4. The MLR model with preceding activity classification showed the overall best performance in terms of MAE with a value of 15.2 ± 6.3%. A perfect activity classification would improve the MAE to 14.6 ± 6.1%. The correlation coefficient between the measured EE and the estimated EE was *R* = 0.92 (*p* < 0.001). In general, all EE estimation models showed a better performance for paraparetic compared to tetraparetic participants, and for women compared to men. All MLR based models underestimated the EE in general, while all ANN-based models overestimated the EE, which is reflected in the MSE. A closer look into the estimation accuracies of the different activity classes revealed that the EE estimation accuracy was similar for the sedentary, the low-intensity, and the walking class, but worse for the high-intensity class compared to the others. A complete overview of the MAE of the different classes can be found in Table S2.

**Table 4:** Evaluation of the different EE estimation models developed within this study, which were either based on a multi-linear regression (MLR) or based on artificial neural network (ANN).

**Model description**	**Mean absolute error (MAE)**						**Mean signed error (MSE)**		**Maximum error**	
	**Overall**		**Tetraparetic**	**Paraparetic**	**Men**	**Women**	**Overall**		**Overall**	
	**[%]**	**[kcal/day]**	**[%]**	**[%]**	**[%]**	**[%]**	**[%]**	**[kcal/day]**	**[%]**	**[kcal/day]**
MLR direct	18.6 ± 7.6	917 ± 478	20.4 ± 7.3	17.8 ± 7.8	19.1 ± 6.5	17.7 ± 9.8	−4.5 ± 12.3	−506 ± 633	53.4	3,766
MLR class known	14.6 ± 6.1	711 ± 398	16.6 ± 8.6	13.6 ± 4.4	15.3 ± 6.8	13.3 ± 4.2	−3.8 ± 8.1	−346 ± 420	50.6	3,335
MLR class estimated	15.2 ± 6.3	744 ± 425	17.2 ± 8.7	14.3 ± 4.8	15.9 ± 7.1	14.0 ± 4.8	−3.4 ± 8.9	−378 ± 486	56.1	3,577
ANN direct	21.8 ± 8.8	915 ± 407	24.8 ± 10.1	20.2 ± 7.8	22.7 ± 8.7	19.9 ± 9.1	8.8 ± 14.2	53 ± 640	47.3	1,603
ANN class known	17.0 ± 7.8	756 ± 320	18.1 ± 9.9	16.4 ± 6.8	17.1 ± 8.1	16.8 ± 7.7	4.9 ± 11.6	5 ± 519	44.6	1,453
ANN class estimated	17.7 ± 8.3	790 ± 376	19.5 ± 10.0	16.8 ± 7.4	17.8 ± 8.3	17.5 ± 8.7	5.1 ± 12.0	0 ± 552	45.5	1,546

*Similar to the REE estimation, the mean absolute error (MAE) in percent was used as a criterion for optimizing the models. Note that the results in this table were calculated across all activities and subjects*.

**Figure 4:**
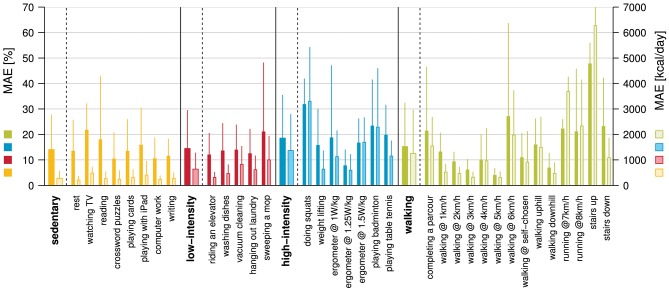
Mean absolute error (MAE) in percent (dark colors) and kcal/day (bright colors) for the EE estimation using the MLR based model with preceding activity classification. The overall MAE for this model was 15.2 ± 6.3%.

For the best EE estimation model, i.e., MLR model with preceding activity classification, the MAEs for all activity classes and single activities are presented in Figure 4. The Bland-Altman comparing the measured EE with the estimated EE using the MLR model with preceding activity classification is can be found in Figure S2 and the corresponding equivalence test in Figure S3.

### Energy Cost of Physical Activity

**Figure 5:**
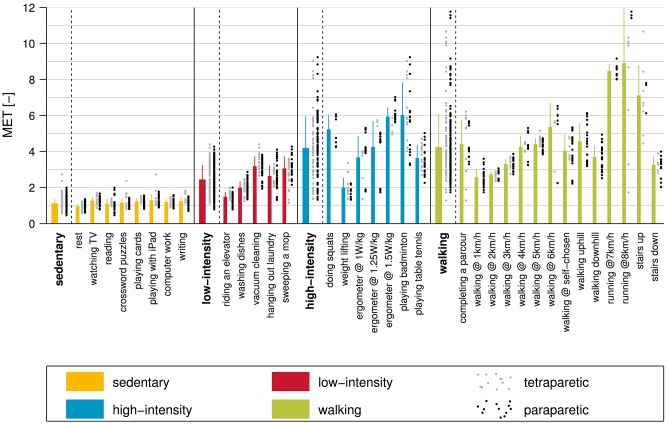
The measured metabolic equivalent of task (MET) for all activities and activity classes included in this study. The different bars represent the mean ± standard deviation and the single values for paraparetic and tetraparetic participants are presented in black and gray, respectively.

**Figure 6:**
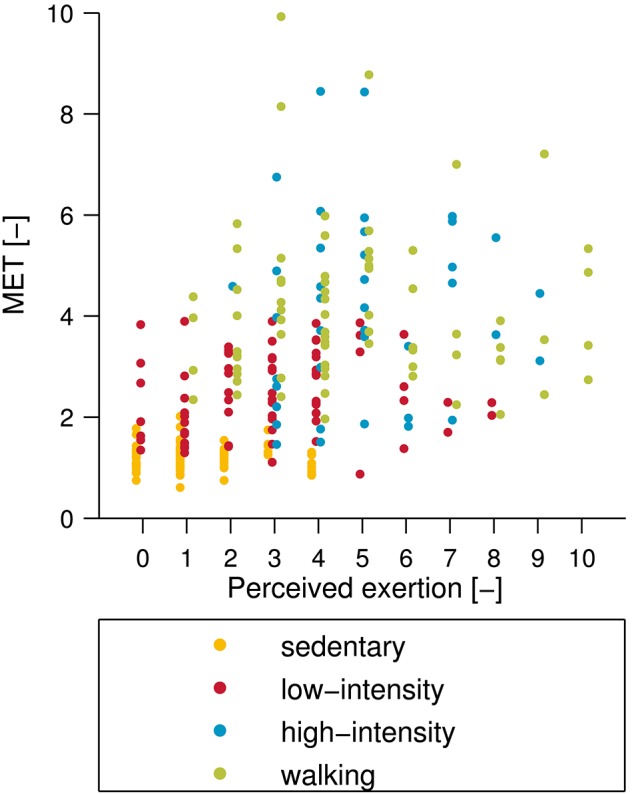
Comparison between subjective perceived exertion and measured metabolic equivalent of task (MET). Note that the subjective perceived exertion was assessed using an 11-point numeric rating scale where 0 represents no exertion and 10 represents maximum exertion. The Spearman rank correlation between perceived exertion and MET was *R* = 0.60 (*p* < 0.001).

The measured METs, for the different activity classes and the single activities, are presented in Figure 5. The average MET for the sedentary class was 1.1 ± 0.3, with the lowest average MET of 0.9 ± 0.2 for rest, and the highest average MET of 1.3 ± 0.4 for playing games on an iPad. The low-intensity class had an average MET of 2.4 ± 0.8 with riding an elevator showing the lowest MET value (1.5 ± 0.3) and vacuum cleaning showing the highest (3.2 ± 0.6). The average MET for the high-intensity class was 4.2 ± 1.8, with average MET values ranging from 2.0 ± 0.6 (weight lifting) to 6.0 ± 1.8 (playing badminton). The walking class showed the broadest spectrum of intensities, ranging from an average MET value of 2.5 ± 0.5 for walking at 1 km/h to 8.8 ± 3.3 for running at 8 km/h. The average MET value for this class was 4.2 ± 1.9. The Spearman rank correlation coefficient between measured MET and perceived exertion was *R* = 0.60 (*p* < 0.001) (Figure 6).

### Translation of Activity Recommendations

To translate the activity recommendations for the non-disabled population to daily activity goals for ambulatory individuals with an iSCI, the measured EE, as well as manually labeled numbers of steps, were used. The average sensitivity for the step detection compared to the manually labeled numbers of step was 85.8 ± 23.2%, and the positive predictive value was 93.7 ± 13.6%. The translation of 30 min of moderate physical activity per day resulted in a recommendation of 1,672–3,715 steps per day depending on the calculation basis and the walking speed. Based on an additional EE of 300 kcal for 10,000 steps reported for the non-disabled population, this daily step goal corresponded to 8,291 steps in ambulatory individuals with an iSCI. The overview of the different translations of activity recommendations can be found in Table 5.

**Table 5:** Translation of different activity guidelines and recommendation for the non-disabled population to daily step goals for ambulatory individuals with an incomplete spinal cord injury (iSCI).

	**Calculation**	**Walking speed**	**EE**	**Time**	**Goal: number of steps**	**Cadence**	**REE**	**measured EE**	**additional EE**
**Recommendation**	**Basis**	**[km/h]**	**[kcal/task]**	**[min]**	**[-]**	**[steps/min]**	**[kcal/day]**	**[kcal/day]**	**[kcal/day]**
	30 min	3	84		2,648	88	1,557	5,579	4,023
	30 min	4	141		3,137	105	1,814	8,566	6,752
	30 min	5	127		3,376	113	1,653	7,737	6,085
	30 min	6	160		3,715	124	1,895	9,859	7,694
30 min of moderate physical	**30 min**	**Average 4.3**^**a**^	**120**		**3,126**	**104**	**1,685**	**7,422**	**5,737**
activity per day	75 kcal	3		27	2,369	88	1,557	5,579	4,023
	75 kcal	4		16	1,672	105	1,814	8,566	6,752
	75 kcal	5		18	1,997	113	1,653	7,737	6,085
	75 kcal	6		14	1,738	124	1,895	9,859	7,694
	**75 kcal**	**Average 4.3**^**a**^		**19**	**1,961**	**104**	**1,685**	**7422**	**5737**
60 min of moderate physical activity per day	**60 min**	**Average 4.3**^**a**^	**239**		**6,251**	**104**	**1,685**	**7,422**	**5,737**
	**150 kcal**	**Average 4.3**^**a**^		**38**	**3,922**	**104**	**1,685**	**7,422**	**5,737**
10,000 steps
	**10,000 steps**	**Average 3.3**^**b**^	**362**	**110**		**91**	**1,641**	**6,359**	**4,718**
	300 kcal	1		173	9,724	56	1,477	3,971	2,494
	300 kcal	2		134	10,170	76	1,661	4,894	3,234
	300 kcal	3		107	9,477	88	1,557	5,579	4,023
	300 kcal	4		64	6,689	105	1,814	8,566	6,752
	300 kcal	5		71	7,989	113	1,653	7,737	6,085
	300 kcal	6		56	6,953	124	1,895	9,859	7,694
	**300 kcal**	**Average 3.3**^**b**^		**92**	**8,291**	**91**	**1,641**	**6,359**	**4,718**
1,000 min of moderate									
physical activity per week^c^	**569 kcal**	**Average 4.3**^**a**^		**143**	**14,872**	**104**	**1,685**	**7,422**	**5,737**
(143 min per day)									

*Note that values in bold are average values over different walking speeds. The average values including all walking speeds equivalent to a moderate intensity activity, i.e., walking between 3 and 6 km/h, are labeled with (a) and average values including all walking speeds, i.e., walking 1–6 km/h, are labeled with a(b). This guideline is based on the work of Thompson et al. (75) (c)*.

## Discussion

In this work, we present an accurate method to estimate EE from wearable sensor recordings in ambulatory individuals with an iSCI. In addition, we translated popular and scientifically informed activity recommendations for the non-disabled population to easily understandable activity recommendations for ambulatory individuals with iSCI. Hereafter, the different EE estimation models with the different analysis steps, i.e., REE estimation, activity classification, and EE estimation, are discussed in detail. Subsequently, the translation of the activity recommendations is reviewed, and the measured EE is compared to literature in order to validate our calculation basis for the translations.

### Updated Harris-Benedict Equations Provide the Best REE Estimate

An accurate estimation of REE is a principal requirement for any EE estimation as REE accounts for ~70% of the total daily EE in chronic SCI (77) while all EE estimation models developed in this study relied on the REE as model input. Therefore, it is obvious, that an inaccurate estimation of the REE would result in a poor EE estimation. Fortunately, all REE estimation models showed satisfying results with an MAE ranging from 10.5 to 13.0%, whereby the updated Harris-Benedict equation (54) showed the best results. These results are in line with previous studies. In our study with the wheelchair-bound participants, the updated Harris-Benedict equations showed also the best results, yet with an MAE, which was ~4% higher than in the current study (30). The higher MAE can be attributed to the fact that body composition, especially muscle mass, is reduced in wheelchair-bound individuals with SCI, due to complete or partial paralysis of skeletal muscles below the NLI (78) and/or due to non-use of the lower extremities. Other studies investigating the accuracy of existing equations showed an overestimation of REE by 5–32% (79–82) in individuals with an SCI.

While the present study showed similar REE estimation errors as previous studies, our estimates tend to underestimate the true REE while in the other studies an overestimation was seen. The difference might come from two sources: First, these studies included mostly individuals with a complete lesion while our study investigated individuals with incomplete lesions. Second, in our study, all participants were able to walk, which has most likely not been the case in the other studies. In the present study, the MSE for the updated Harris-Benedict equation is indeed negative (−3.3%), but close to 0%. This might suggest that the iSCI ambulatory individuals included in this study are similar to the non-disabled population in terms of body composition. Therefore, it is a valid approach to use the updated Harris-Benedict equation for the estimation of the REE in our EE estimation model.

One possibility to further improve the estimation accuracy of REE is to apply prediction equations developed specifically for SCI individuals as recently proposed by Nightingale et al. (80). So far, this approach has been developed for motor complete SCI individuals only and would need to be adapted to the population of incomplete SCI as well.

### Activity Classification Results in Excellent Accuracy

In a previous study with SCI wheelchair users, we have shown improved EE estimation accuracy by ~3% (30) when using separate estimation models for different activity classes. Similar results have also been shown for the non-disabled population (51, 83). In order to apply different EE estimation models for different activity classes, an accurate activity classifier is needed. In this study, we used a kNN classifier, which showed very good classification performance with an overall accuracy of 95.6%. The overall classification accuracy exceeded comparable models developed for the incomplete SCI population by 4% (84) and 6.7% (85). However, these models were built on a single waist-mounted sensor only but included only six activities (lying, sitting, walking, standing, wheeling, and stair climbing) compared to the 12 tasks investigated in the presented study, which makes a comparison challenging.

The sensitivity for the different activity classes was very high for the sedentary and the walking class. The results can be explained by the fact that both activities have very specific and regular movement patterns, i.e., almost no movement at all and very regular movement patterns, which can easily be separated by the classifier. In contrast, the low-intensity and high-intensity class show partly very smooth transitions, which can be challenging for the classifier. For that reason, it is not surprising that the sensitivity was slightly lower for the low-intensity class (94.6%) and reasonably lower for the high-intensity class (81.0%). The single activity showing the worst sensitivity was doing squats (37.5%) followed by playing table tennis (72.5%), weight lifting (75.0%), training on the bicycle ergometer at 1.25 W per kg body weight (75.0%), and playing badminton (82.1%). There are two main reasons why activities were misclassified. First, in some activities, e.g., “doing squats” or “weight lifting,” the EE can be high (MET>3) while movements are minimal or very slow. This cannot be captured through IMUs but could possibly be overcome by combining sensor measurements with heart rate measurements, galvanic skin response measurements, or near body temperature measurements as done in previous studies (14, 26). However, in our previous study with wheelchair users, adding the heart rate data did not significantly increase the EE estimation accuracy and was therefore omitted in the current study. Second, activities such as “playing badminton” or “table tennis” include rallies that can partly be very exhausting. However, these intensive phases are repeatedly interrupted by less intensive phases, e.g., while collecting shuttles or balls, which in turn can directly change the classification. Actually, this is not a true misclassification, as these activities are intermittent, and longer, less intensive phases can indeed be classified as low-intense activities. All other activities had sensitivities over 89% and 20 out of 34 activities had no misclassification. Generally, the kNN classifier presented in this work showed overall a very good classification accuracy and can, therefore, be used for the following EE estimation. Since this approach focused on broader activity classes instead of single activities, it makes this kNN classifier well-suited for real-world applications and likely also for activities not included in this study. However, to confirm this, a separate validation in the free-living environment including new activities is needed.

### EE Estimation Accuracy Show Similar Performance to Models for the Non-disabled Population

All EE estimation models developed within this study showed satisfactory results, with MAEs ranging from 14.6 to 21.8%. The overall best EE estimation model, which can be applied in a real-world application, is the MLR-based model with preceding activity classification showing an MAE of 15.2%. Note, that the EE estimation model with an MAE of 14.6% assumes a perfect activity classification, which was only evaluated in order to see the effect of misclassification on the EE estimation. To the best of our knowledge, no dedicated EE estimation model for ambulatory individuals with an iSCI exists. Thus, we can only compare our EE estimation models to other populations. The EE estimation models for wheelchair users with an SCI showed similar or slightly worse accuracies than the present study (21–23, 86). In comparison with our previous EE estimation model for wheelchair users with an SCI, the EE estimation for ambulatory individuals with an iSCI is slightly worse (MAE of 15.2 vs. 14.4%). This might indicate that the population of ambulatory individuals with an iSCI is more heterogeneous than the population of wheelchair users with an SCI. This heterogeneity is for example reflected in the widely varying performance during the 6MWT in which a participant with almost no gait deficits achieved a distance of 744 m while another participant, using two forearm crutches, only achieved 137 m. Heterogeneity is further induced by the fact that a part of the population used walking aids, which has been shown to increase EE additionally (34). Another factor leading to the slightly decreased EE estimation compared to the model for wheelchair users is the decreased accuracy in activity classification in the present model. Classification of activities in SCI individuals using a wheelchair could be achieved with a higher accuracy (97.9%) than in incomplete SCI individuals with the ability to walk (96.6%).

The result of EE estimation models designed for conditions other than SCI with pathological gait can either not directly be compared due to different evaluation metrics used or they show slightly worse results than the present study (37–40). The differences in estimation accuracies might be explained by the fact that in the present study, the information of multiple sensors and therefore, body locations were available. When comparing our EE estimation model with that of commercial activity trackers for the non-disabled population (12), our model performs among the best despite the heterogeneity of our ambulatory individuals with an iSCI. Again, the results can be explained by the number of sensors used for the EE estimation, as multiple sensors can help to capture functional deficits from different body locations. EE estimation models developed for the non-disabled population using information from multiple sensors (and therefore, body locations) showed slightly better performance than the model presented in this study (87). Nevertheless, the MLR model with the preceding activity classification presented in this work shows a very good estimation accuracy considering the heterogeneity of the included population.

The different EE estimation models developed within this study, i.e., ANN and MLR based models, both benefited from the preceding activity classification. In fact, the MAE decreased by around 4%. This value is slightly better than values reported for the inclusion of preceding activity classification in the EE estimation models for the wheelchair-bound individuals (decrease in MAE: 0.3% MLR based model, 3.2% ANN-based model) (30). This observation can be explained by the fact that in the current study, four activity classes were used in contrast to the study with the wheelchair-bound individuals with only three activity classes. Additionally, the activity classes in the previous study with wheelchair users depended on upper-limb movements only and were thus similar in terms of body movements. Therefore, the activity classification only marginally improved the EE estimation model. This is in contrast to the present study, where the different activity classes can be completely different in terms of movement patterns, i.e., sedentary class vs. walking class.

In general, the improvement in EE estimation accuracy with preceding activity classification is in line with results from previous studies in the non-disabled population (12, 51). Surprisingly, the MLR based EE estimation model outperformed the ANN-based model in terms of MAE by 2–3%, which might be due to the heterogeneity of the present population. It is likely that outliers might be less heavily weighted using MLRs. These outliers can be assumed to be the few individuals with walking aids included, showing different movement patterns compared to individuals without walking aids. By including more participants, the ANN-based EE estimation model might improve the estimation accuracy and might also outperform the MLR-based EE estimation model.

Having a closer look at the different activity classes, we can see that the EE estimation worked well for the sedentary, the low-intensity, and the walking class. The MAE for the single activity classes when the MLR-based model with preceding activity classification was used, lied between 14.0 and 15.2%. Only the high-intensity class showed a worse MAE (18.5%). In return, this class was the one, which benefitted most from a preceding activity classification (improvement of ~7%). The reason why the high-intensity class showed, in general, the worst MAE is due to the different activities included. Five out of seven PA in this class are so-called weight-loading activities in which the external load cannot be captured through the sensors. Thereby the EE estimation accuracy decreases, which has already been shown in other EE estimation studies (88, 89).

When investigating the features selected for the different EE estimation models, we realize that features from all sensors, i.e., accelerometer, gyroscope, and altimeter, were included. This is identical to the study with the wheelchair-dependent individuals, where all sensors have been included as well (30). Taking a closer look at the best EE estimation model, i.e., MLR model with preceding activity classification, we can see that the only high-level feature included was the laterality of the feet (included in the sedentary and the low-intensity class). This could indicate that the laterality of the feet might be a potential indicator for gait deficits (kind of quality measure), similar to the laterality of the wrist, which is considered as a measure of functional deficits of the upper-limbs (66, 90). From the demographic and anthropometric features and assessment scores, only one feature was included, namely the body weight in the walking class. However, demographic and anthropometric data such as gender, age, weight, and height are also included in the REE and thus indirectly included as features. An examination on how many features are provided from which sensor location (only MLR models) showed that features from the chest sensor were the most prominent, with a total of eight occurrences. One explanation of why the chest sensor contributed so many features to the EE estimation model might be that the chest sensor can capture movements from the upper- and the lower-limbs at the same time. Furthermore, features from the lower extremities (feet: eight features and ankles: two features) were included more often than features from the upper extremities (wrists: two features). This is not surprising, because the activity-dependent EE is mostly dominated by the lower extremities due to the larger muscle groups. In summary, the MLR based model with preceding activity classification is a very good model for the estimation of EE, which can well be applied in real-world settings. The present approach has mainly been developed for applications in research in which a high accuracy is required. Further work is needed to investigate the feasibility of reduced number of sensors, which is needed for application in daily life.

### Energy Cost of Physical Activities Found in Ambulatory Individuals With an iSCI Are in Agreement With Values Reported in the Literature

First, we would like to demonstrate that participants included in this study are representative of the population of ambulatory individuals with an iSCI. For this purpose, the MET values obtained in this study were compared to MET values from the literature. Two studies have reported MET values for ambulatory individuals with iSCI. Collins et al. determined METs for 27 different physical activity (91). While most of the activities were performed in a sitting position, e.g., sitting in a wheelchair, two of the activities included walking. The converted MET (from SCI MET) for level walking at a self-chosen pace was 3.7 for tetraparetic participants and 3.6 for paraparetic participants which is in the same range as we have seen in the current study (4.0 ± 0.9). For climbing stairs, however, METs were slightly different between Collins et al. (4.7) and the current study (7.1 ± 1.7), a difference which may likely be attributed to the difference in the much larger slope of the stairs in the present study and the number of participants (30 in the present study, two in Collins et al.'s study). Two additional activities, which were performed in a sitting position, are present in both studies, namely deskwork and weight lifting. The reported MET values are very similar to the MET values of the present study, namely 1.0 MET for deskwork (present study: 1.2 ± 0.2 MET for computer work and 1.2 ± 0.3 MET for writing) and 2.1–2.4 MET (depending on the NLI) for weight training (present study: 2.0 ± 0.6 MET). The remaining activities which were present in both studies, i.e., playing table tennis, doing the laundry, vacuuming, and washing dishes, cannot be compared directly as it is unknown if participants in the study by Collins et al. were standing or sitting during the activities. The second study reporting MET values for ambulatory individuals with an iSCI included four activities, which were also present in the current study, i.e., supported and unsupported sitting, standing, and walking (92). Reported MET values are very similar to those of the present study, i.e., supported and unsupported sitting (both 1.0 MET) vs. reading (1.1 ± 0.3 MET) and watching TV (1.3 ± 0.2 MET) in the present study, and standing (1.2 MET) vs. riding an elevator (1.5 ± 0.3 MET) although riding an elevator can include single steps and interacting with an interface. The last common activity was walking at self-chosen speed, where an average of 3.4 MET was reported (range 3.0–4.5) by Dekker and coworkers compared to 4.0 ± 0.9 MET in the present study. In summary, it can be stated that MET values reported in this study match the values reported in literature. Thus, we are confident that it is appropriate to use the present MET values to translate activity recommendations.

### A Minimum of 2,000–3,000 Steps per Day Should Be Achieved by Ambulatory Individuals With an iSCI

For the translation of activity recommendations, we first considered recommendations from global health institutions and second the popular recommendation of 10,000 steps a day.

Most of the activity recommendations from global institutions recommend beside strength training, flexibility training and skill training, 30 min of moderate activity on at least 5 days per week (6). The WHO and the HHS recommendations are kept broader with 150 min of additional moderate-intensity aerobic PA per week (7–9). These recommendations, however, can be satisfied by engaging in moderate PA five times a week for 30 min. Other research groups go even one step further and suggest 30 min of moderate PA every day (73, 74). We focused on the last recommendation of 30 min of moderate PA per day for our translation of activity recommendations. However, the obtained values can easily be adapted to other recommendations, e.g., PA, on only 5 days per week. In the current study, we aimed to translate the aforementioned activity recommendation for the non-disabled population to a simple and understandable activity recommendation for ambulatory individuals with an iSCI. Therefore, all activity recommendations were translated into daily step goals. The activity recommendation of 30 min of moderate PA per day was translated by using 30 min of moderate PA or by using 75 kcal of additional EE during moderate PA [approximately corresponding to 30 min of moderate PA (74)] as calculation basis. Moderate PA is defined by a MET value between 3 and 5.9 (93). Based on our data, this corresponds to a walking speed of 3–6 km/h (MET of 3.3–5.4, Figure 5).

When using 30 min as calculation basis, the resulting daily step goals increased with increasing walking speed due to an increasing cadence (2,648 steps for 3 km/h and 3,715 steps for 6 km/h). The measured values of cadence are in line with findings reported by Awai and coworkers (94). Having a closer look at the additional energy expended during these 30 min of moderate PA, we measured 84–160 kcal, which lies above the 75 kcal which is assumed to be an equivalent of 30 min of moderate-intensity PA in non-disabled ambulatory individuals (74). The differences in EE can be explained as follow: First, other sources suggest 75–150 kcal for 30 min of moderate PA, and we decided to go for the value at the lower end. Second, some of the included participants had a relatively high REE, which results in an increased activity-dependent EE. Third, although the cadence and step length are similar between ambulatory individuals with an iSCI and neurologically intact individuals (94), the EE is increased in pathological gait, as has been shown for other neurological conditions (34). For these reasons, it is not surprising that the EE is slightly increased in ambulatory individuals with iSCI.

When using the additional 75 kcal spent in moderate PA as a calculation basis, the proposed step goal would be lower, around 2,000 steps per day. The time required for reaching this additional 75 kcal would lie between 14 and 27 min depending on speed, with an average at around 20 min. These 20 min of moderate PA corresponds to the activity recommendation proposed by Ginis et al. for cardiorespiratory fitness and muscle strength benefits (together with three sets of strength exercise two times per week) even if this recommendation should be followed only two times per week (42). In order to follow the cardio-metabolic health guideline for adults with an SCI suggesting 30 min of moderate to vigorous intensity aerobic exercise three times per week (42), 3,000 steps per day three times per week should be accomplished. For the compliance with the 60 min of moderate PA recommendation (74), which should bring additional health benefits such as preventing weight gain, our data can easily be extrapolated, e.g., 150 kcal of additional EE (corresponding to 60 min of moderate PA) would correspond to ~4,000 steps.

The last translation involves the 10,000 steps per day activity recommendation. Based on the data collected in this present study, participants would burn an additional 362 kcal on average when walking 10,000 steps. This is slightly more than the suggested 300 kcal corresponding to 10,000 steps per day in the non-disabled population. In order to reach the 300 kcal of additional EE, ambulatory individuals with an iSCI would have to achieve between 6,000 and 10,000 steps per day depending on their walking speed. The average lies at around 8,000 steps. The ~20% reduced number of steps can be explained by the increased energy requirement during pathological gait as outlined above. Interestingly, the highest step recommendation (10'170 steps/day) would be given when participants were walking at around 2 km/h. This could mean that ambulatory individuals with an iSCI show the most ergonomic gait pattern at this speed. This hypothesis is underpinned by the fact that ambulatory individuals with an iSCI have a preferred walking speed of around 2 km/h (94). However, walking at 2 km/h is, according to our measurements, not a PA of moderate intensity (MET = 2.7 ± 0.2) but the adherence to 8,000 steps per day at preferred walking speed would certainly break a sedentary lifestyle.

In summary, it can be stated that at an ambulatory individual with an iSCI should walk at least 2,000–3,000 steps per day at a moderate intensity, defined as an exertion score of 5–6 on a 0–10 scale (9); in order to fulfill the activity recommendation of the WHO (8) to have a significant health impact and reduce mortality. Nevertheless, 8,000 steps per day (equivalent to 10,000 steps per day in the non-disabled population) would certainly be beneficial. Note that these activity recommendations are only valid if no sporting activities are performed, as these would count toward the activity goal. Furthermore, it has to be confirmed that the proposed daily step goal of 8,000 steps per day, without prescribing an intensity, is sufficient for a health-promoting effect. For individuals with severe gait disabilities, the proposed PA goal might be too high, and it might be better to follow the recommendations proposed by Ginis et al. (42). However, with the data provided in this study, the activity recommendations could easily be converted to other recommendations.

### Limitations

The study has several limitations. First, the number of participants using walking aids is relatively low (*n* = 5) and not all kinds of walking aids have been included in the study, e.g., we had no quad cane and walker. Validation is required showing that the developed algorithm can also be applied when using other walking aids or when using a combination of walking aids. Second, the proposed algorithm does not take fitness or the lifestyle of an individual into account, which potentially could improve the EE estimation accuracy. Third, although the different tasks were performed as similar as possible to real-world applications (no constraints on how to perform a task), both, the classification algorithm and EE estimation algorithm, have first to be validated under real-world settings. This also involves the inclusion of types of PA that were not included in the current study. Fourth, although ambulatory individuals with an iSCI have a similar body composition to the non-disabled population, the energy expenditure during gait is increased and the variability between individuals might be higher than in the non-disabled population. Especially for that reason, it has first to be shown that activity recommendations for the non-disabled population can be translated one-to-one or if they have to be adapted to specific sub-populations such as individuals with more severe functional deficits.

## Conclusion

In this work, we present the first dedicated EE estimation model for ambulatory individuals with an iSCI, which is based on recordings of wearable sensors. By using state of the art wearable sensors, the EE can be continuously recorded over multiple days in a non-obstructive way under real-world situations. Together with the translated and comprehensive activity recommendations, this methodology can be a key part for a potential activity tracker, which is specifically designed for ambulatory individuals with an iSCI, allowing for promotion of a healthy lifestyle without discouraging users due to recommendations that are too high. Additionally, the EE estimation model developed in this study in combination with the EE estimation model, which we have developed for wheelchair users with an SCI (30), allows tracking the EE and also training intensity over the entire rehabilitation process in SCI. This could potentially lead to new insights into how PA and especially the intensity of PA affects functional recovery.

## Data Availability Statement

The datasets for this study will not be made publicly available because of confidentiality requirements. Requests to access the datasets should be directed to WP [werner.popp@hest.ethz.ch].

## Ethics Statement

All subjects gave written informed consent in accordance with the Declaration of Helsinki. The protocol was approved by the local ethics committee of the canton of Zurich (KEK-ZH Nr. 2013-0202).

## Author Contributions

WP, SS, CS, RG, and AC designed the study. WP, SS, JB, and PB collected the experimental data. JB and PB labeled the data and performed the preprocessing. WP analyzed the data. WP, SS, JB, PB, CS, RG, and AC interpreted the results, revised the manuscript, and approved the final version.

### Conflict of Interest

The authors declare that the research was conducted in the absence of any commercial or financial relationships that could be construed as a potential conflict of interest.

## References

[1] Tudor-Locke C Bassett DR Jr. How many steps/day are enough? Sports Med. (2004) 34:1–8. 10.2165/00007256-200434010-00001 14715035

[2] Tudor-Locke C Craig CL Aoyagi Y Bell RC Croteau KA De Bourdeaudhuij I. How many steps/day are enough? For older adults and special populations. Int J Behav Nutr Phys Activity. (2011) 8:80. 10.1186/1479-5868-8-80 21798044 PMC3169444

[3] Morgan AL Tobar DA Snyder L. Walking toward a new me: the impact of prescribed walking 10,000 steps/day on physical and psychological well-being. J Phys Activity Health. (2010) 7:299–307. 10.1123/jpah.7.3.299 20551485

[4] Wilde BE Sidman CL Corbin CB. A 10,000-step count as a physical activity target for sedentary women. Res Q Exerc Sport. (2001) 72:411–4. 10.1080/02701367.2001.10608977 11770790

[5] Le-Masurier GC Sidman CL Corbin CB. Accumulating 10,000 steps: does this meet current physical activity guidelines? Res Q Exerc Sport. (2003) 74:389–94. 10.1080/02701367.2003.10609109 14768840

[6] Haskell WL Lee I-M Pate RR Powell KE Blair SN Franklin BA. Physical activity and public health: updated recommendation for adults from the American college of sports medicine and the American heart association. Circulation. (2007) 116:1081. 10.1161/CIRCULATIONAHA.107.185649 17671237

[7] US Department of Health Human Services Physical Activity Guidelines for Americans. Washington, DC: Department of Health and Human Services (2008).

[8] World Health Organization Global Recommendations on Physical Activity for Health. Geneva: WHO Press (2010). 26180873

[9] Piercy KL Troiano RP Ballard RM Carlson SA Fulton JE Galuska DA. The physical activity guidelines for Americans. JAMA. (2018) 320:2020–8. 10.1001/jama.2018.14854 30418471 PMC9582631

[10] Ainsworth BE Haskell WL Herrmann SD Meckes N Bassett DR Jr Tudor-Locke C. 2011 Compendium of physical activities: a second update of codes and MET values. Med Sci Sports Exerc. (2011) 43:1575–81. 10.1249/MSS.0b013e31821ece12 21681120

[11] Ainsworth BE Haskell WL Whitt MC Irwin ML Swartz AM Strath SJ. Compendium of physical activities: an update of activity codes and MET intensities. Med Sci Sports Exerc. (2000) 32 (9; SUPP/1):S498–504. 10.1097/00005768-200009001-00009 10993420

[12] Dannecker KL Sazonova NA Melanson EL Sazonov ES Browning RC. A comparison of energy expenditure estimation of several physical activity monitors. Med Sci Sports Exerc. (2013) 45:2105. 10.1249/MSS.0b013e318299d2eb 23669877 PMC3800491

[13] Ferguson T Rowlands AV Olds T Maher C. The validity of consumer-level, activity monitors in healthy adults worn in free-living conditions: a cross-sectional study. Int J Behav Nutr Phys Activity. (2015) 12:42. 10.1186/s12966-015-0201-9 25890168 PMC4416251

[14] Chowdhury EA Western MJ Nightingale TE Peacock OJ Thompson D. Assessment of laboratory and daily energy expenditure estimates from consumer multi-sensor physical activity monitors. PLoS ONE. (2017) 12:e0171720. 10.1371/journal.pone.0171720 28234979 PMC5325221

[15] Cooper C Gross A Brinkman C Pope R Allen K Hastings S. The impact of wearable motion sensing technology on physical activity in older adults. Exp Gerontol. (2018) 112:9–19. 10.1016/j.exger.2018.08.002 30103025 PMC6436091

[16] Wang JB Cadmus-Bertram LA Natarajan L White MM Madanat H Nichols JF. Wearable Sensor/Device (Fitbit One) and SMS text-messaging prompts to increase physical activity in overweight and obese adults: a randomized controlled trial. Telemed J E Health. (2015) 21:782–92. 10.1089/tmj.2014.0176 26431257 PMC4842945

[17] Coughlin SS Stewart J. Use of consumer wearable devices to promote physical activity: a review of health intervention studies. J Environ Health Sci. (2016) 2. 10.15436/2378-6841.16.1123 28428979 PMC5395205

[18] Washburn R Hedrick B. Descriptive epidemiology of physical activity in university graduates with locomotor disabilities. Int J Rehab Res. (1997) 20:275–88. 10.1097/00004356-199709000-00004 9331577

[19] Cragg JJ Noonan VK Krassioukov A Borisoff J. Cardiovascular disease and spinal cord injury: results from a national population health survey. Neurology. (2013) 81:723–8. 10.1212/WNL.0b013e3182a1aa68 23884034 PMC3776463

[20] Buchholz AC Martin Ginis KA Bray SR Craven BC Hicks AL Hayes KC. Greater daily leisure time physical activity is associated with lower chronic disease risk in adults with spinal cord injury. Appl Physiol Nutr Metab. (2009) 34:640–7. 10.1139/H09-050 19767799

[21] Hiremath SV Ding D Farringdon J Cooper RA. Predicting energy expenditure of manual wheelchair users with spinal cord injury using a multisensor-based activity monitor. Arch Phys Med Rehab. (2012) 93:1937–43. 10.1016/j.apmr.2012.05.004 22609119

[22] Hiremath SV Intille SS Kelleher A Cooper RA Ding D. Estimation of energy expenditure for wheelchair users using a physical activity monitoring system. Arch Phys Med Rehab. (2016) 97:1146–1153.e1. 10.1016/j.apmr.2016.02.016 26976800

[23] Nightingale T Walhin J Thompson D Bilzon J. Predicting physical activity energy expenditure in wheelchair users with a multisensor device. BMJ Open Sport Exerc Med. (2015) 1:bmjsem-2015-000008. 10.1136/bmjsem-2015-000008 27900111 PMC5117017

[24] Garcia-Masso X Serra-Ano P Garcia-Raffi L Sanchez-Perez E López-Pascual J Gonzalez L. Validation of the use of Actigraph GT3X accelerometers to estimate energy expenditure in full time manual wheelchair users with spinal cord injury. Spinal Cord. (2013) 51:898–903. 10.1038/sc.2013.85 23999111

[25] Hiremath SV Ding D editors. Regression equations for RT3 activity monitors to estimate energy expenditure in manual wheelchair users. In: Engineering in Medicine and Biology Society, EMBC, 2011 Annual International Conference of the IEEE. IEEE (2011). 10.1109/IEMBS.2011.6091714 22256036

[26] Nightingale TE Walhim J-P Thompson D Bilzon JL. Predicting physical activity energy expenditure in manual wheelchair users. Med Sci Sports Exerc. (2014) 46:1849–58. 10.1249/MSS.0000000000000291 25134004

[27] Nightingale TE Walhin J-P Thompson D Bilzon JLJ. Influence of accelerometer type and placement on physical activity energy expenditure prediction in manual wheelchair users. PLoS One. (2015) 10:e0126086. 10.1371/journal.pone.0126086 25955304 PMC4425541

[28] Hiremath SV Ding D. Evaluation of activity monitors in manual wheelchair users with paraplegia. J Spinal Cord Med. (2011) 34:110–7. 10.1179/107902610X12911165975142 21528634 PMC3066485

[29] Tsang K. Evaluation of custom energy expenditure models for SenseWear armband in manual wheelchair users. J Rehab Res Dev. (2015) 52:793. 10.1682/JRRD.2014.01.0188 26745837

[30] Popp WL Richner L Brogioli M Wilms B Spengler CM Curt AEP. Estimation of energy expenditure in wheelchair-bound spinal cord injured individuals using inertial measurement units. Front Neurol. (2018) 9:478. 10.3389/fneur.2018.00478 30018586 PMC6037746

[31] Jayaraman C Mummidisetty CK Mannix-Slobig A Koch LM Jayaraman A. Variables influencing wearable sensor outcome estimates in individuals with stroke and incomplete spinal cord injury: a pilot investigation validating two research grade sensors. J Neuroeng Rehab. (2018) 15:19. 10.1186/s12984-018-0358-y 29534737 PMC5850975

[32] Schaffer SD Holzapfel SD Fulk G Bosch PR. Step count accuracy and reliability of two activity tracking devices in people after stroke. Physiother Theory Pract. (2017) 33:788–96. 10.1080/09593985.2017.1354412 28777710

[33] Elsworth C Dawes H Winward C Howells K Collett J Dennis A. Pedometer step counts in individuals with neurological conditions. Clin Rehab. (2009) 23:171–5. 10.1177/0269215508098895 19164404

[34] Waters RL Mulroy S. The energy expenditure of normal and pathologic gait. Gait Posture. (1999) 9:207–31. 10.1016/S0966-6362(99)00009-0 10575082

[35] Waters RL Adkins R Yakura J Vigil D. Prediction of ambulatory performance based on motor scores derived from standards of the American Spinal Injury Association. Arch Phys Med Rehab. (1994) 75:756–60. 10.1016/0003-9993(94)90034-5 8024420

[36] Ladlow P Nightingale TE McGuigan MP Bennett AN Phillip RD Bilzon JLJ. Predicting ambulatory energy expenditure in lower limb amputees using multi-sensor methods. PLoS ONE. (2019) 14:e0209249. 10.1371/journal.pone.0209249 30703115 PMC6354995

[37] Sandroff BM Motl RW Suh Y. Accelerometer output and its association with energy expenditure in persons with multiple sclerosis. J Rehab Res Dev. (2012) 49:467. 10.1682/JRRD.2011.03.0063 22773205

[38] Sandroff BM Riskin BJ Agiovlasitis S Motl RW. Accelerometer cut-points derived during over-ground walking in persons with mild, moderate, and severe multiple sclerosis. J Neurol Sci. (2014) 340:50–7. 10.1016/j.jns.2014.02.024 24635890

[39] Furlanetto KC Bisca GW Oldemberg N Sant'Anna TJ Morakami FK Camillo CA. Step counting and energy expenditure estimation in patients with chronic obstructive pulmonary disease and healthy elderly: accuracy of 2 motion sensors. Arch Phys Med Rehab. (2010) 91:261–7. 10.1016/j.apmr.2009.10.024 20159131

[40] Manns PJ Haennel RG. Sensewear Armband and stroke: validity of energy expenditure and step count measurement during walking. Stroke Res Treat. (2012) 2012:247165. 10.1155/2012/247165 23094200 PMC3475303

[41] Mandigout S Lacroix J Ferry B Vuillerme N Compagnat M Daviet J-C. Can energy expenditure be accurately assessed using accelerometry-based wearable motion detectors for physical activity monitoring in post-stroke patients in the subacute phase? Eur J Prev Cardiol. (2017) 24:2009–16. 10.1177/2047487317738593 29067851

[42] Ginis KAM van der Scheer JW Latimer-Cheung AE Barrow A Bourne C Carruthers P. Evidence-based scientific exercise guidelines for adults with spinal cord injury: an update and a new guideline. Spinal Cord. (2018) 56:308. 10.1038/s41393-017-0017-3 29070812

[43] Ginis KM Hicks A Latimer A Warburton D Bourne C Ditor D. The development of evidence-informed physical activity guidelines for adults with spinal cord injury. Spinal Cord. (2011) 49:1088. 10.1038/sc.2011.63 21647164

[44] van der Scheer JW Martin Ginis KA Ditor DS Goosey-Tolfrey VL Hicks AL West CR. Effects of exercise on fitness and health of adults with spinal cord injury: a systematic review. Neurology. (2017) 89:736–45. 10.1212/WNL.0000000000004224 28733344

[45] Kirshblum SC Burns SP Biering-Sorensen F Donovan W Graves DE Jha A. International standards for neurological classification of spinal cord injury (revised 2011). J Spinal Cord Med. (2011) 34:535–46. 10.1179/204577211X13207446293695 22330108 PMC3232636

[46] Catz A Itzkovich M. Spinal Cord Independence Measure: comprehensive ability rating scale for the spinal cord lesion patient. J Rehabil Res Dev. (2007) 44:65. 10.1682/JRRD.2005.07.0123 17551859

[47] Kalsi-Ryan S Beaton D Curt A Duff S Popovic MR Rudhe C. The graded redefined assessment of strength sensibility and prehension: reliability and validity. J Neurotr. (2012) 29:905–14. 10.1089/neu.2010.1504 21568688

[48] Kalsi-Ryan S Curt A Verrier MC Fehlings MG. Development of the Graded Redefined Assessment of Strength, Sensibility and Prehension (GRASSP): reviewing measurement specific to the upper limb in tetraplegia. J Neurosurg. (2012) 17(Suppl. 1):65–76. 10.3171/2012.6.AOSPINE1258 22985372

[49] van Hedel HJ Wirz M Dietz V. Assessing walking ability in subjects with spinal cord injury: validity and reliability of 3 walking tests. Arch Phys Med Rehabil. (2005) 86:190–6. 10.1016/j.apmr.2004.02.010 15706542

[50] Riener R. The Cybathlon promotes the development of assistive technology for people with physical disabilities. J Neuroeng Rehabil. (2016) 13:49. 10.1186/s12984-016-0157-2 27246601 PMC4886429

[51] Staudenmayer J Pober D Crouter S Bassett D Freedson P. An artificial neural network to estimate physical activity energy expenditure and identify physical activity type from an accelerometer. J Appl Physiol. (2009) 107:1300–7. 10.1152/japplphysiol.00465.2009 19644028 PMC2763835

[52] Garnotel M Bastian T Romero-Ugalde H-M Maire A Dugas J Zahariev A. Prior automatic posture and activity identification improves physical activity energy expenditure prediction from hip-worn triaxial accelerometry. J Appl Physiol. (2017) 124:780–90. 10.1152/japplphysiol.00556.2017 29191980

[53] Harris JA Benedict FG. A biometric study of human basal metabolism. Proc Natl Acad Sci USA. (1918) 4:370–3. 10.1073/pnas.4.12.370 16576330 PMC1091498

[54] Roza AM Shizgal HM. The Harris Benedict equation reevaluated: resting energy requirements and the body cell mass. Am J Clin Nutr. (1984) 40:168–82. 10.1093/ajcn/40.1.168 6741850

[55] Mifflin MD St Jeor ST Hill LA Scott BJ Daugherty SA Koh Y. A new predictive equation for resting energy expenditure in healthy individuals. Am J Clin Nutr. (1990) 51:241–7. 10.1093/ajcn/51.2.241 2305711

[56] Müller MJ Bosy-Westphal A Klaus S Kreymann G Lührmann PM Neuhäuser-Berthold M. World Health Organization equations have shortcomings for predicting resting energy expenditure in persons from a modern, affluent population: generation of a new reference standard from a retrospective analysis of a German database of resting energy expenditure. Am J Clin Nutr. (2004) 80:1379–90. 10.1093/ajcn/80.5.1379 15531690

[57] Curone D Bertolotti GM Cristiani A Secco EL Magenes G. A real-time and self-calibrating algorithm based on triaxial accelerometer signals for the detection of human posture and activity. IEEE Trans Inform Technol Biomed. (2010) 14:1098–105. 10.1109/TITB.2010.2050696 20483689

[58] Ravi N Dandekar N Mysore P Littman ML editors. Activity Recognition From Accelerometer Data. Pittsburgh, PA: AAAI (2005).

[59] Herren R Sparti A Aminian K Schutz Y. The prediction of speed and incline in outdoor running in humans using accelerometry. Med Sci Sports Exerc. (1999) 31:1053–9. 10.1097/00005768-199907000-00020 10416569

[60] Bao L Intille SS editors. Activity recognition from user-annotated acceleration data. In: International Conference on Pervasive Computing. Berlin; Heidelberg: Springer (2004). 10.1007/978-3-540-24646-6_1

[61] Stikic M Huynh T Van Laerhoven K Schiele B editors. ADL recognition based on the combination of RFID and accelerometer sensing. In: 2008 Second International Conference on Pervasive Computing Technologies for Healthcare. Tampere: IEEE (2008). 10.1109/PCTHEALTH.2008.4571084

[62] Leuenberger K Gonzenbach R Wiedmer E Luft A Gassert R editors. Classification of stair ascent and descent in stroke patients. In: Wearable and Implantable Body Sensor Networks Workshops (BSN Workshops), 2014 11th International Conference on. Zurich: IEEE (2014). 10.1109/BSN.Workshops.2014.10

[63] Moncada-Torres A Leuenberger K Gonzenbach R Luft A Gassert R. Activity classification based on inertial and barometric pressure sensors at different anatomical locations. Physiol Meas. (2014) 35:1245. 10.1088/0967-3334/35/7/1245 24853451

[64] Popp WL Brogioli M Leuenberger K Albisser U Frotzler A Curt A. A novel algorithm for detecting active propulsion in wheelchair users following spinal cord injury. Med Eng Phys. (2016) 38:267–74. 10.1016/j.medengphy.2015.12.011 26868046

[65] Pratama AR Hidayat R editors. Smartphone-based pedestrian dead reckoning as an indoor positioning system. In: System Engineering and Technology (ICSET), 2012 International Conference on. Bandung: IEEE (2012). 10.1109/ICSEngT.2012.6339316

[66] Brogioli M Popp WL Albisser U Brust AK Frotzler A Gassert R. Novel sensor technology to assess independence and limb-use laterality in cervical spinal cord injury. J Neurotr. (2016) 33:1950–7. 10.1089/neu.2015.4362 27025797

[67] Bailey RR Klaesner JW Lang CE. An accelerometry-based methodology for assessment of real-world bilateral upper extremity activity. PLoS ONE. (2014) 9:e103135. 10.1371/journal.pone.0103135 25068258 PMC4113366

[68] Bailey RR Klaesner JW Lang CE Quantifying real-world upper-limb activity in non-disabled adults and adults with chronic stroke. Neurorehabil Neural Repair. (2015) 29:969–78. 10.1177/1545968315583720 25896988 PMC4615281

[69] Leuenberger K Gonzenbach R Wachter S Luft A Gassert R A method to qualitatively assess arm use in stroke survivors in the home environment. Med Biol Eng Comp. 2016:1–10. 10.1007/s11517-016-1496-7 PMC5222943 27106757

[70] Tofallis C Least squares percentage regression. J Modern Appl Stat Methods. (2009) 7:526–34. 10.2139/ssrn.1406472

[71] Hagan MT Menhaj MB. Training feedforward networks with the Marquardt algorithm. IEEE Trans Neural Networks. (1994) 5:989–93. 10.1109/72.329697 18267874

[72] Nguyen D Widrow B editors. Improving the learning speed of 2-layer neural networks by choosing initial values of the adaptive weights. In: Neural Networks, 1990, 1990 IJCNN International Joint Conference. San Diego, CA: IEEE (1990). 10.1109/IJCNN.1990.137819

[73] Blair SN LaMonte MJ Nichaman MZ. The evolution of physical activity recommendations: how much is enough? Am J Clin Nutr. (2004) 79:913S−20S. 10.1093/ajcn/79.5.913S 15113739

[74] Wilson T Bray GA Temple NJ Struble MB Nutrition Guide for Physicians. Totowa, NJ: Springer (2010). 10.1007/978-1-60327-431-9

[75] Thompson D Batterham AM Peacock OJ Western MJ Booso R Feedback from physical activity monitors is not compatible with current recommendations: a recalibration study. Prev Med. (2016) 91:389–94. 10.1016/j.ypmed.2016.06.017 27330025 PMC5061550

[76] Choi BC Pak AW Choi JC. Daily step goal of 10,000 steps: a literature review. Clin Invest Med. (2007) 30:146–51. 10.25011/cim.v30i3.1083 17716553

[77] Nightingale TE Williams S Thompson D Bilzon JL. Energy balance components in persons with paraplegia: daily variation and appropriate measurement duration. Int J Behav Nutr Phys Activity. (2017) 14:132. 10.1186/s12966-017-0590-z 28950900 PMC5615439

[78] Price M. Energy expenditure and metabolism during exercise in persons with a spinal cord injury. Sports Med. (2010) 40:681–96. 10.2165/11531960-000000000-00000 20632738

[79] Buchholz AC McGillivray CF Pencharz PB. Differences in resting metabolic rate between paraplegic and able-bodied subjects are explained by differences in body composition. Am J Clin Nutr. (2003) 77:371–8. 10.1093/ajcn/77.2.371 12540396

[80] Nightingale TE Gorgey AS. Predicting basal metabolic rate in men with motor complete spinal cord injury. Med Sci Sports Exerc. (2018) 50:1305–12. 10.1249/MSS.0000000000001548 29315167

[81] Andersen RE Sweet SN Reid RE Sydney F Plourde H. Accuracy of two generic prediction equations and one population-specific equation for resting energy expenditure in individuals with spinal cord injury. Can J Diet Pract Res. (2018) 79:1–6. 10.3148/cjdpr-2018-021 30014708

[82] Buchholz AC Pencharz PB. Energy expenditure in chronic spinal cord injury. Curr Opin Clin Nutr Metab Care. (2004) 7:635–9. 10.1097/00075197-200411000-00008 15534431

[83] Bonomi AG Plasqui G Goris AH Westerterp KR. Improving assessment of daily energy expenditure by identifying types of physical activity with a single accelerometer. J Appl Physiol. (2009) 107:655–61. 10.1152/japplphysiol.00150.2009 19556460

[84] Albert MV Azeze Y Courtois M Jayaraman A. In-lab versus at-home activity recognition in ambulatory subjects with incomplete spinal cord injury. J Neuroeng Rehabil. (2017) 14:10. 10.1186/s12984-017-0222-5 28166824 PMC5294819

[85] Sok P Xiao T Yohannes A Jayaraman A Albert MV Activity recognition for incomplete spinal cord injury subjects using hidden markov models. IEEE Sensors J. (2018) 18:6369–74. 10.1109/JSEN.2018.2845749

[86] Hiremath S Ding D Farringdon J Vyas N Cooper R. Physical activity classification utilizing SenseWear activity monitor in manual wheelchair users with spinal cord injury. Spinal Cord. (2013) 51:705–9. 10.1038/sc.2013.39 23689386

[87] Liu S Gao RX John D Staudenmayer JW Freedson PS. Multisensor data fusion for physical activity assessment. IEEE Trans Biomed Eng. (2012) 59:687–96. 10.1109/TBME.2011.2178070 22156943

[88] Swartz AM Strath SJ Bassett DR O Brien WL King GA Ainsworth BE. Estimation of energy expenditure using CSA accelerometers at hip and wrist sites. Med Sci Sports Exerc. (2000) 32(9; SUPP/1):S450–S6. 10.1097/00005768-200009001-00003 10993414

[89] Brage S Brage N Franks PW Ekelund U Wong M-Y Andersen LB. Branched equation modeling of simultaneous accelerometry and heart rate monitoring improves estimate of directly measured physical activity energy expenditure. J Appl Physiol. (2004) 96:343–51. 10.1152/japplphysiol.00703.2003 12972441

[90] Brogioli M Popp WL Schneider S Albisser U Brust AK Frotzler A. Multi-day recordings of wearable sensors are valid and sensitive measures of function and independence in human spinal cord injury. J Neurotr. (2016) 34:1141–8. 10.1089/neu.2016.4583 27533063

[91] Collins EG Gater D Kiratli J Butler J Hanson K Langbein WE. Energy cost of physical activities in persons with spinal cord injury. Med Sci Sports Exerc. (2010) 42:691–700. 10.1249/MSS.0b013e3181bb902f 19952846

[92] Dekker B Verschuren O Balemans AC Baart N Tubbing F van Koppenhagen CF Energy expenditure and muscle activity during lying, sitting, standing, and walking in people with motor-incomplete spinal cord injury. Spinal Cord. (2018) 2018:1 10.1038/s41393-018-0167-y 29955089

[93] Pate RR Pratt M Blair SN Haskell WL Macera CA Bouchard C. Physical activity and public health: a recommendation from the Centers for Disease Control and Prevention and the American College of Sports Medicine. JAMA. (1995) 273:402–7. 10.1001/jama.1995.03520290054029 7823386

[94] Awai L Bolliger M Ferguson AR Courtine G Curt A. Influence of spinal cord integrity on gait control in human spinal cord injury. Neurorehabil Neural Repair. (2016) 30:562–72. 10.1177/1545968315600524 26428035 PMC4818198

