# Supplementary material for: Wearable Sensors in Ambulatory Individuals With a Spinal Cord Injury: From Energy Expenditure Estimation to Activity Recommendations
Source: Front Neurol. 2019 Nov 1;10:1092. doi: 10.3389/fneur.2019.01092 (PMC6838774; doi:10.3389/fneur.2019.01092)
Supplement: Supplementary file 1 [file Data_Sheet_1.pdf]

Statistical features	acceleration magnitude	gyroscope magnitude	gyroscope Z-axis*	altitude
mean	o	o	o	
median	o	o	o	
variance	o	o	o	o
standard deviation	o	o	o	o
IQR	o	o	o	o
RMS	o	o	o	
kurtosis	o	o	o	o
AC	o	o	o	
percentiles {1, 3, 5, 25, 75, 95, 97 99}	o	o	o	
**Additional features**				
anthropometric and demographic data, assessment scores				
numbers of step, distance walked, variance in stride time				
lateralities (feet, wrists, ankles, upper vs. lower limbs)				
correlations (acceleration magnitudes between: wrists, ankles, feet)				
altitude changes				

*Only calculated for the ankle sensor data. Angular velocity of the z-axis corresponds to a rotation in the sagittal plane.

Table S1: Overview of all extracted features considered for the kNN classifier. For developing the EE estimation models, the estimated REE using the updated Harris-Benedict equation was included as an additional feature. Note that features from the gyroscope z-axis were only extracted from the ankle sensors.

	Mean absolute error (MAE)			
**Model description**	sedentary [%]	low intensity [%]	high intensity [%]	walking [%]
MLR direct	16.5	17.0	25.2	19.2
MLR class known	13.8	14.0	15.3	15.1
MLR class estimated	14.0	14.5	18.5	15.2
ANN direct	22.8	17.8	22.7	22.1
ANN class known	14.7	15.9	17.9	19.9
ANN class estimated	15.4	16.1	19.0	20.3

Table S2: Mean absolute errors (MAE) for the EE estimation of the different classes. The overall estimation, including all activity classes together can be found in Table 3.

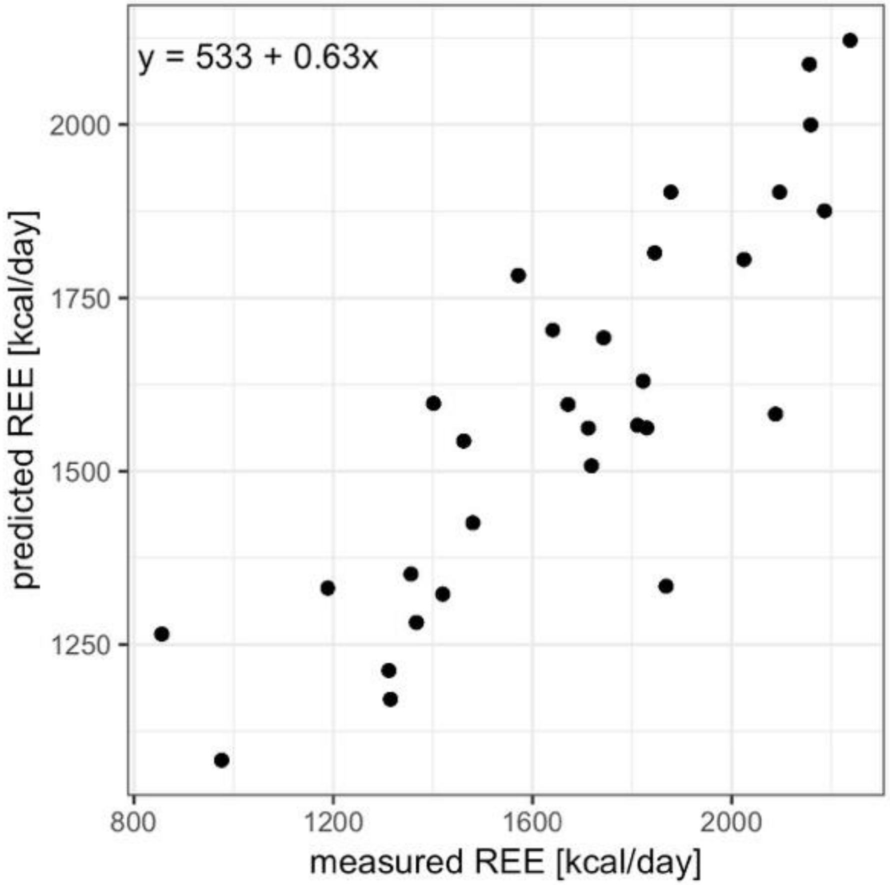

Figure S1: Predicted REE using the updated Harris-Benedict equation (y) plotted against measured REE (x) with the calculated regression formula.

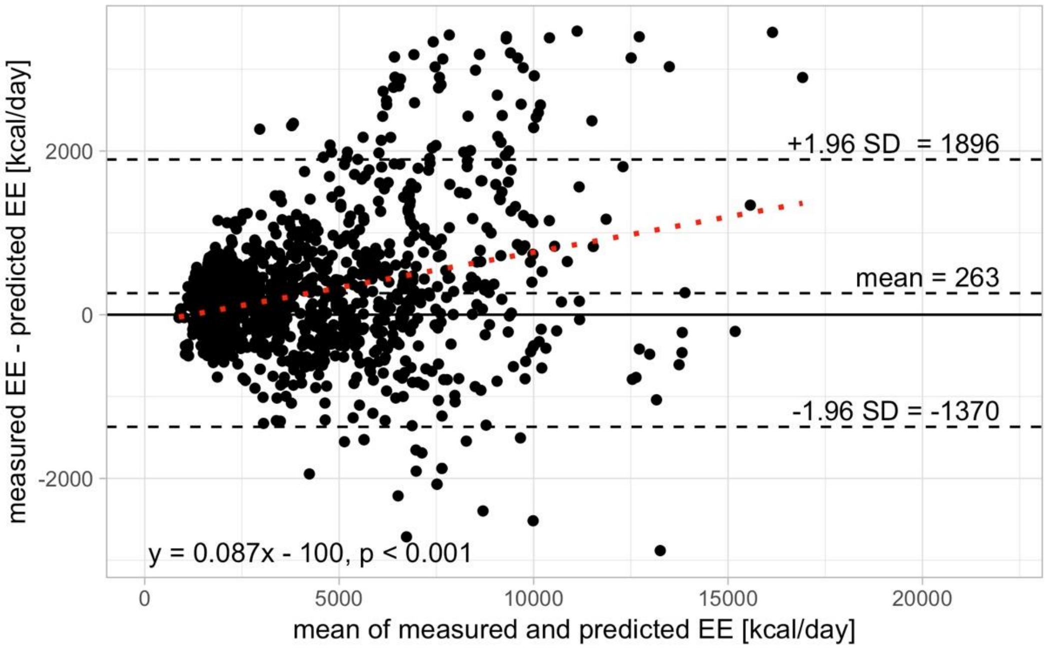

Figure S2: Bland-Altman plot of the measured EE compared to the predicted EE using the MLR model with preceding activity classification. The mean bias was 263 kcal/day with 95% limits of agreement of 1896 kcal/day and -1370 kcal/day. A significant slope was found (slope: 0.087, p-value < 0.001).

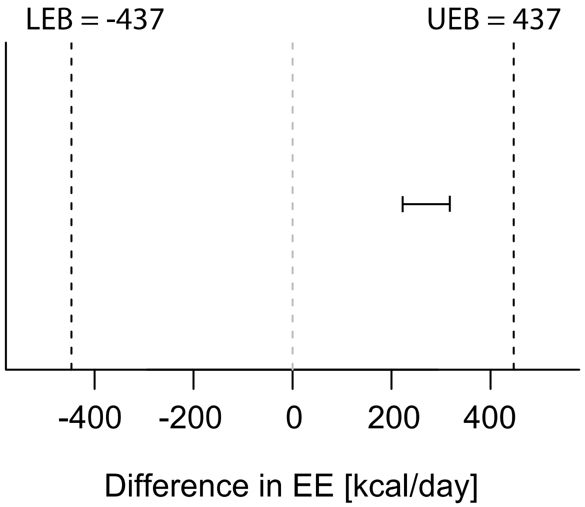

Figure S3: Paired equivalence testing between measured EE and predicted EE using the MLR based model with preceding activity classification. The horizontal bar indicates the 95% two one-sided confidence interval around the estimated mean of the differences (263 [224; 302] kcal/day). Equivalence zone (dashed vertical lines) was set to 10% of the measured mean resulting in a lower equivalence bound (LEB) of -437 kcal/day and an upper equivalence bound (UEB) of 437 kcal/day. Equivalence can be assumed with a p-value < 0.001 (t(1235) = -7.337).
